# The Effect of WS_2_ Nanosheets on the Non-Isothermal Cold- and Melt-Crystallization Kinetics of Poly(l-lactic acid) Nanocomposites

**DOI:** 10.3390/polym13132214

**Published:** 2021-07-05

**Authors:** Mohammed Naffakh, Pablo Rica, Carmen Moya-Lopez, José Antonio Castro-Osma, Carlos Alonso-Moreno, Diego A. Moreno

**Affiliations:** 1 Escuela Técnica Superior de Ingenieros Industriales, Universidad Politécnica de Madrid (ETSII-UPM), José Gutiérrez Abascal 2, 28006 Madrid, Spain; pablo.rica.mozo@alumnos.upm.es (P.R.); diego.moreno@upm.es (D.A.M.); 2 Facultad de Farmacia, Universidad de Castilla-La Mancha (FF-UCLM), Avda. Dr. José María Sánchez Ibáñez s/n, 02008 Albacete, Spain; carmen.moya-lopez-pelaez@univ-lorraine.fr (C.M.-L.); JoseAntonio.castro@uclm.es (J.A.C.-O.); carlos.amoreno@uclm.es (C.A.-M.); 3 LMOPS, CentraleSupelec, University of Lorraine, 2 Rue E. Belin, 57070 Metz, France; 4 Centro Regional de Investigaciones Biomédicas, Unidad NanoCRIB, 02008 Albacete, Spain

**Keywords:** 2D-TMDCs WS_2_, PLLA, nanomaterials, morphology, crystallization, melting

## Abstract

In the present work, hybrid nanocomposite materials were obtained by a solution blending of poly(l-lactic acid) (PLLA) and layered transition-metal dichalcogenides (TMDCs) based on tungsten disulfide nanosheets (2D-WS_2_) as a filler, varying its content between 0 and 1 wt%. The non-isothermal cold- and melt-crystallization and melting behavior of PLLA/2D-WS_2_ were investigated. The overall crystallization rate, final crystallinity, and subsequent melting behavior of PLLA were controlled by both the incorporation of 2D-WS_2_ and variation of the cooling/heating rates. In particular, the analysis of the cold-crystallization behavior of the PLLA matrix showed that the crystallization rate of PLLA was reduced after nanosheet incorporation. Unexpectedly for polymer nanocomposites, a drastic change from retardation to promotion of crystallization was observed with increasing the nanosheet content, while the melt-crystallization mechanism of PLLA remained unchanged. On the other hand, the double-melting peaks, mainly derived from melting–recrystallization–melting processes upon heating, and their dynamic behavior were coherent with the effect of 2D-WS_2_ involved in the crystallization of PLLA. Therefore, the results of the present study offer a new perspective for the potential of PLLA/hybrid nanocomposites in targeted applications.

## 1. Introduction

The dependency on petrochemical-based polymers has drastically increased over the last few years because the properties of these versatile macrostructures, such as strength or flexibility, make them suitable for many applications. However, the chemical inertness of these polymers to chemical and biological degradation is an important limitation from an environmental point of view. In this very context, polymers derived from biomass are considered as promising candidates to replace them. In this regard, polylactide (PLA) is produced from renewable resources by condensation reactions of lactic acid monomers or by ring-opening polymerization (ROP) of lactide monomers with metal catalysts [1]. The latter favors an increase in polymeric molecular weights and control over the polymerization process. In particular, poly-l-lactide (PLLA) is the product resulting from the polymerization of L-lactide and is being extensively studied with the aim of replacing conventional petrochemical-based polymers for use in either the industrial packaging field or the biocompatible/bioabsorbable medical device market [2,3]. In comparison to PLA, PLLA has better mechanical properties based on its crystallinity and is comparable to polyethylene, polypropylene and polystyrene. Although PLLA can be processed on standard converting equipment with minimal modifications, its unique material properties must be taken into consideration in order to optimize the conversion of PLLA to molded parts, films, foams, and fibers [4]. In this way, understanding the crystallization behavior is particularly crucial to control PLLA’s degradation rate, thermal resistance as well as optical, mechanical and barrier properties [5].

The addition of fillers is one of the most cost-effective methods to improve the physical properties of biopolymeric materials. The most common fillers are nucleating agents, low-cost engineering polymers and nanoparticles. The former increases the crystallinity of the material in a short period of thermal treatment and the latter reduces the overall cost of the biopolymeric materials. The amount of crystallinity in the hybrid systems has a significant impact on the functional, mechanical, structural, and physical properties of the final product. Despite many experiments to understand the effect of additives on crystallization, the results are still contradictory. The discrepancy is likely related to several factors and none can be ruled out: the difference in the thermal conductivity of the filler and polymer matrix; the nucleation efficiency (NE) of the filler; its state of dispersion within the matrix; and the potential existence of mechanisms of interfacial crystallization such as epitaxy and transcrystallization [6,7,8,9]. In addition, NE is strongly dependent on the nanofiller morphology, its surface energy, roughness and crystalline structure as well as on the filler’s ability to form the critical nucleus [10,11]. In this regard, Jabbarzadeh has recently reported the crystallization origin in nanocomposite polymers [12]. Results of large-scale molecular dynamic simulations revealed that crystallinity is affected by nanoparticle size and its volume fraction. However, thorough understanding of the dynamic of these systems, including the mobilities of the different constituents, also remains an extremely difficult task [13,14]. In particular, the production of high-quality nanocomposites requires good particle dispersion and strong interaction and adhesion between the nanofiller and polymer matrix. 

The use of layered transition-metal dichalcogenide nanostructures (TMDCs), such as molybdenum disulfide (MoS_2_) and tungsten disulfide (WS_2_), which are high-band gap semiconductors with 0D, 1D and 2D structural anisotropy, is particularly interesting as a proposal of improvements [15,16,17,18,19,20]. TMDCs exhibit many technologically important and scientifically interesting properties, often linked to their anisotropy. In particular, monolayer MoS_2_ [15], with high surface areas and superb thermal stability and excellent mechanical properties, has recently been reported to efficiently enhance the mechanical and barrier properties of various kinds of polymeric materials, whilst not affecting their electrical insulation properties [16,17,18]. In this particular case, the use of environmentally friendly and biocompatible inorganic TMDCs allowed the production of novel biopolymer-based nanocomposite materials (Bio-PNCs 1D-TMDCs WS_2_) [21,22,23,24]. Notable among these, 1D-WS_2_ exhibited higher nucleation efficiency, which significantly improved the melt and cold-crystallization behavior of PLLA. The incorporation of only 0.1 wt% of 1D-WS_2_ allowed the crystallization of PLLA at a cooling rate of 10 °C/min, and the crystallization peak temperature (*T*_p_) increased by up to 17 °C. This value corresponds to the highest value observed hitherto for PLLA formulations using nano-sized fillers (e.g., MWCNT, SCWCNT, C60, GO) [24]. Additionally, 1D-WS_2_ can act as an efficient nucleating agent as well as a reinforcing filler in PLLA/hydroxyapatite (HA) blends obtained by a conventional melt blending technique, thus remarkably enhancing their chemical and thermal stability, mechanical performance under dry and simulated body fluid (SBF) conditions as well as tribological properties. The improvements are more pronounced in the hybrid nanocomposite with the highest HA content, likely due to a synergistic effect of both micro and nano-fillers on the matrix performance [25]. On the other hand, the effects of 2D-WS_2_ on the melt-crystallization and biodegradation of melt-processable PLLA nanocomposites have recently been investigated. Promising results for the biodegradation behavior of a standard PLLA commercial grade have been recently reported [26], especially the addition of 2D-WS_2_ into the PLLA matrix to facilitate the enzymatic degradation of poorly biodegradable PLLA using a promising strain of actinobacteria, *Lentzea waywayandensis*.

The present research continues work in this field based on the use of 2D-TMDCs WS_2_ to produce new hybrid nanocomposite materials through simple solution blending of PLLA. Herein, hybrid nanocomposites were successfully obtained by ROP using living organometallic initiators. The influence of the 2D-TMDCs WS_2_ concentration on the non-isothermal cold- and melt-crystallization behavior of PLLA polymer was analyzed. In particular, the knowledge of the cold-crystallization behavior of the polymer chains under confined conditions provides valuable information regarding the processing and use of PLLA.

## 2. Experimental Section

### 2.1. Solution Polymerization of PLLA

All manipulations were performed under nitrogen, using standard Schlenk techniques. Solvents were pre-dried over sodium wire (toluene, n-hexane and THF) and distilled under nitrogen from sodium (toluene and THF) or sodium–potassium alloy (n-hexane). Deuterated solvents were stored over activated 4 Å molecular sieves and degassed by several freeze–thaw cycles. 1H and 13C NMR spectra were recorded on a Varian Inova FT-500 spectrometer (VARIAN Inc, CA, USA) and referenced to the residual deuterated solvent (Figures S2 and S3, Supporting Information). AlEt3 was purchased from Sigma-Aldrich, Madrid, Spain, and l-lactide from REX Scientific (REX Scientific, York, UK) L-Lactide was sublimed three times, recrystallized from THF and finally sublimed again prior to use. Initiator 1 was prepared according to literature procedures [27]. Gel permeation chromatography (GPC) measurements were performed on a Polymer Laboratories PL-GPC-220 instrument (Malvern Panalyticalm, Malvern, UK) equipped with a TSK-GEL G3000H (Sigma Aldrich, Madrid, Spain) column and an ELSD-LTII light-scattering detector (Malvern Panalyticalm, Malvern, UK) (Figure S4, Supporting Information). The GPC column was eluted with THF at 50 °C at 1 mL/min and was calibrated using eight monodisperse polystyrene standards in the range 580–48,3000 Da.

Polymerizations of l-LA were performed on a Schlenk line in a flame-dried Schlenk flask equipped with a magnetic stirrer. The Schlenk tubes were charged in the glovebox with the required amount of l-LA and initiator 1 (Figure S1, Supporting Information), separately, and then attached to the vacuum line. The initiator and monomer were dissolved in the appropriate amount of solvent, and temperature equilibration was ensured in both Schlenk flasks by stirring the solutions for 15 min in an oil bath. The solution of initiator was added by syringe to the monomer and polymerization times were measured from that point. Polymerizations were stopped by injecting a solution of acetic acid (5 vol-%) in methanol. Polymers were precipitated in methanol, filtered, dissolved in THF, reprecipitated in methanol, and dried in vacuum to constant weight.

### 2.2. Preparation of PLLA/2D-WS_2_ Nanocomposites

In this study, 2D-WS_2_ nanosheets (width/length ≈ 20–500 nm and thickness ≈ 1 nm) were obtained from ACS Material LLC (Medford, MA, US) and used without chemical modification. Several concentrations of 2D-WS_2_ (0.1, 0.5 and 1.0 wt%) were dispersed in a solution of PLLA in DCM (1g L^−1^) (HPLC grade, Sigma-Aldrich Química SL, Madrid, Spain) and homogenized by mechanical stirring for 10 min. Subsequently, DCM was evaporated off at room temperature while stirring with Ultra-Turrax T 50 Digital Disperser. The PLLA/2D-WS_2_ was dried under vacuum for 48 h.

### 2.3. Characterization Techniques of PLLA/2D-WS_2_ Nanocomposites

Ultra-high field-emission scanning microscopy (FESEM), JEOL-JSM7600F (Tokyo, Japan) and transmission electron microscopy (TEM), JEOL-JEM 2100 (Tokyo, Japan) were used to characterize the morphology and dispersion of 2D-WS_2_ in the PLLA matrix. 

Non-isothermal cold- and melt-crystallization of the neat PLLA and PLLA/2D-WS_2_ nanocomposites were measured using a TA Instrument Discovery Differential Scanning Calorimeter DSC 25 (Waters Chromatography, Madrid, Spain, N_2_ gas, 50 mL/min flow rate). An amount of 2 to 5 mg of each sample was sealed in standard aluminum pans. Data were evaluated using the TRIOS software (Waters Chromatography, Madrid, Spain).

DSC melt-crystallization for each sample was heated to 225 °C and held at this temperature for 5 min to erase their thermal history. Afterwards, cooling cycles from the melt were undertaken for each sample at cooling rates (ϕ_c_) of 1, 2, 5, 10 and 20 °C/min, followed by a heating cycle from 40 to 225 °C at 10 °C/min. On the other hand, the samples for cold-crystallization were first cooled from the melt to 40 °C at a rate of 50 °C/min and then heated to 225 °C at heating rates (ϕ_h_) of 1, 2, 5, 10 and 20 °C/min. The heat evolved during the non-isothermal crystallization was recorded as a function of temperature. The crystallization peak temperature (T_c_) was determined from the minimum of the crystallization exotherm observed during the cooling scan, and the melting temperature (T_m_) and the cold-crystallization peak temperature (T_cc_) were obtained from the maximum of the melting endotherm and the minimum of the crystallization exotherm observed during the heating scan, respectively. The apparent crystallization enthalpy was determined as the area below the transformation curve, and taking the upper and lower limits as the corresponding deviations in the baseline, crystallinity was calculated as follows:

(1) $$\left(1-\lambda \right)=\frac{\Delta H_{c}}{\Delta H_{m}^{0}}$$

where ΔH_c_ is the crystallization enthalpy and ΔH^0^_m_ is the enthalpy of melting for perfect crystals (93 J/g) [28]. On the other hand, the measured rate of heat release is assumed to be proportional to the macroscopic rate of crystallization:

(2) $$\frac{\mathrm{dQ}}{\mathrm{dt}}=Q_{c}\frac{\mathrm{dx}}{\mathrm{dt}}$$

where Q_c_ is the measured heat of crystallization calculated by integration of a DSC peak. The values of Qc can further be used to determine the crystallization rate (dx/dt) as well as the extent of the melt conversion:

(3) $$x\left(t\right)=\frac{1}{Q_{c}}\overset{t}{\underset{0}{\int }}\frac{\mathrm{dQ}}{\mathrm{dt}}\mathrm{dt}$$

The value of x(t) varies from 0 to 1 and represents the degree of conversion. The transformation from temperature to time is performed using a constant cooling rate φ:

(4) $$t=\frac{T_{0}-T}{\varphi }$$

where T is the temperature at time t and T_0_ is the temperature at the start of crystallization.

## 3. Results

### 3.1. Morphology

**Figure 1:**
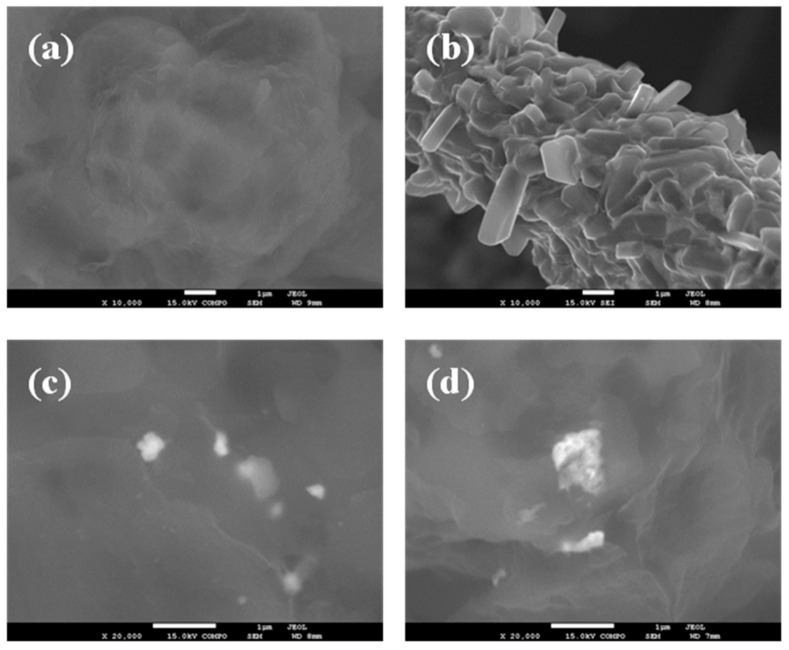
High-resolution SEM micrographs of (**a**) PLLA, (**b**) 2D-WS_2_ and PLLA/2D-WS_2_ nanocomposites with nanofiller loadings of (**c**) 0.1 and (**d**) 1.0 wt%.

**Figure 2:**
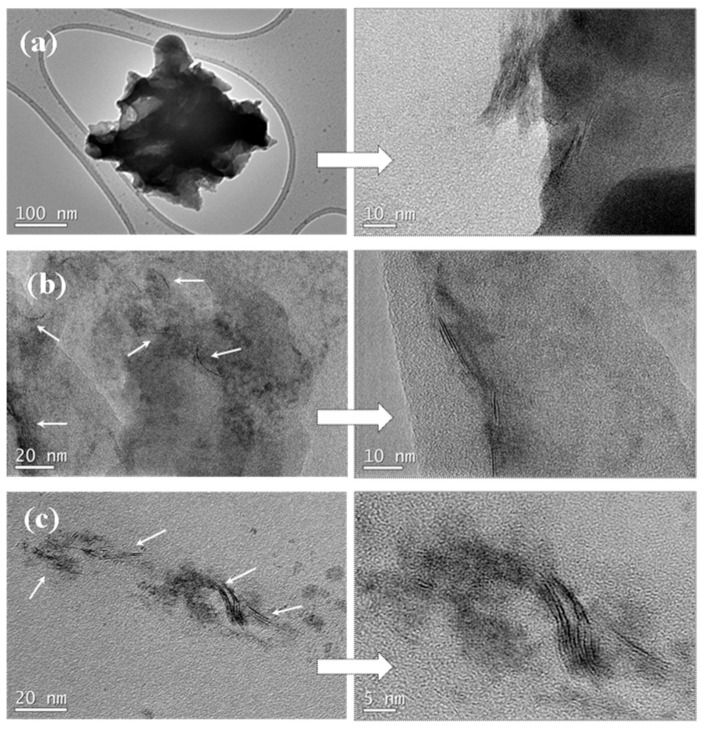
High-resolution SEM micrographs of (**a**) 2D-WS_2_ and PLLA/2D-WS_2_ nanocomposites with nanofiller loadings of (**b**) 0.1 and (**c**) 1.0 wt%.

To obtain information concerning the dispersion and interfacial interactions between the PLLA matrix and WS_2_ nanosheets, the micro-morphology of the cryogenically fractured surfaces of PLLA, the hybrid nanocomposite films, and the neat WS_2_ nanosheets were studied by SEM images (Figure 1). Figure 1a shows a relatively smooth fractured surface for the neat PLLA, whereas a much rougher fractured surface of PLLA hybrid films with 2D-WS_2_ loading of 0.1 and 1 wt% is observed without exhibiting pulled out nor aggregated WS_2_ nanosheets (Figure 1c,d). The well-dispersed 2D-WS_2_ and the formation of stronger interfacial interactions between WS_2_ nanosheets and the PLLA matrix are worthy of note. TEM images support the well-dispersed state of WS_2_ nanosheets within the PLLA matrix without obvious aggregates, mainly shown with individual layers or intercalated structures (Figure 2). In this regard, elaborate methodologies have been previously reported for the synthesis of PLA/MoS_2_-NH_2_ nanocomposites [29]. In our case, the WS_2_ nanosheets exhibited better dispersion and semi-exfoliated structure in the solution samples than the melt-processable counterparts [26]. The good dispersion and strong interfacial adhesion may support a significant stress transfer to reinforce the properties of PLLA nanocomposites.

### 3.2. Non-Isothermal Cold-Crystallization

It is well-known that the physicochemical changes during an exothermic event in DSC are complex and involve multistep processes occurring simultaneously at different rates. In particular, for crystallization from the glassy state, which is governed by chain mobility rather than by nucleation, the diffusion rate of the very entangled longer chains is much smaller than that for the low molecular weight chains, leading to a reduced crystallization rate. Unlike melt-crystallization, in which the motion of polymer chains can be carried out entirely via molecular reptation [30], the polymer chains in the rubbery state complete the corresponding conformational rearrangements via cooperative segmental movements [31].

**Figure 3:**
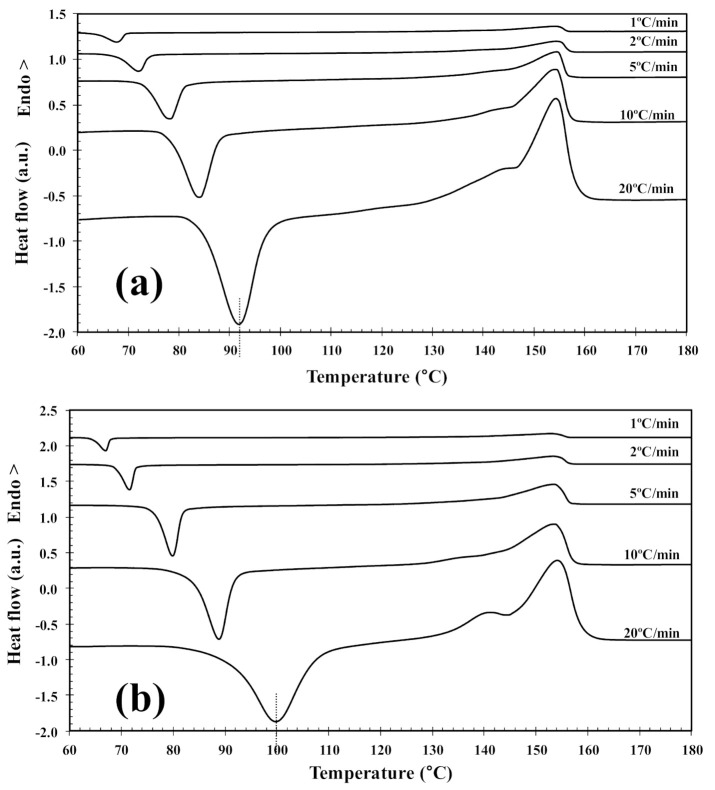
DSC cold-crystallization thermograms of (**a**) PLLA and (**b**) PLLA/2D-WS2 (0.5 wt%).

In order to investigate the cold-crystallization behavior of neat PLLA and PLLA/2D-WS_2_ nanocomposites, DSC curves at different heating rates were obtained to determine the kinetic parameters (i.e., rate constant, crystalline transformation, etc.). Figure 3 shows the DSC heating thermograms for neat PLLA and PLLA/2D-WS_2_ (0.5 wt%) nanocomposites recorded at heating rates of 1, 2, 5, 10 and 20 °C/min after rapid cooling from 225 °C at 50 °C/min, and the specific values of the crystalline parameters of all samples are listed in Table 1. As can be seen, both heating rate and 2D-WS_2_ loading were the two main factors that affected the non-isothermal cold-crystallization and melting behavior of PLLA in the nanocomposites. With increasing heating rate, the crystallization exotherms became broader, and the cold-crystallization peak temperature (T_cc_) shifted to a higher temperature. Moreover, at a given heating rate (for example, 10 °C/min), T_cc_ for neat PLLA was 84.0 °C, whereas T_cc_ for 0.1, 0.5 and 1.0 wt% was found to be 87.0, 88.8, and 89.3 °C, respectively. Such results indicate that the incorporation of 2D-WS_2_ into PLLA decreased its rate of crystallization. This is because the surface of the WS_2_ nanosheets could not easily absorb the PLLA chain segments, which would greatly hinder crystal growth. In particular, when the interparticle free space becomes smaller than the characteristic extended length of the polymer molecule, nanoparticles impede crystallization due to confinement effects. This hypothesis is based on the findings regarding confinement-induced retardation of crystallization, in which equations for critical particle size or volume fraction were proposed [12]. On the other hand, the heating of neat PLLA and PLLA/2D-WS_2_ nanocomposites led to the appearance of complex double-melting peaks. The first peak was observed at 141–145 °C and the other at 154–155 °C (Table 1). The analogous data of double-melting peaks versus heating rate with 2D-WS_2_ concentration as a parameter are also shown in Table 1. In general, it is accepted that the double endothermic peaks are attributed to melting–recrystallization–melting processes of PLLA lamellae. The first endothermic peak is attributed to the melting of thin lamellae formed during the DSC heating process (e.g., cold-crystallization), and the second to the melting of lamellae that are newly formed through the melting–recrystallization of primary thin lamellae occurring at relatively higher temperature [32,33]. In particular, increasing the heating rate allows less time for the crystals to reorganize and re-melting occurs over a lower temperature range. Ideally, the melting of reorganized crystals should completely vanish over a certain heating rate where recrystallization is totally inhibited.

**Figure 4:**
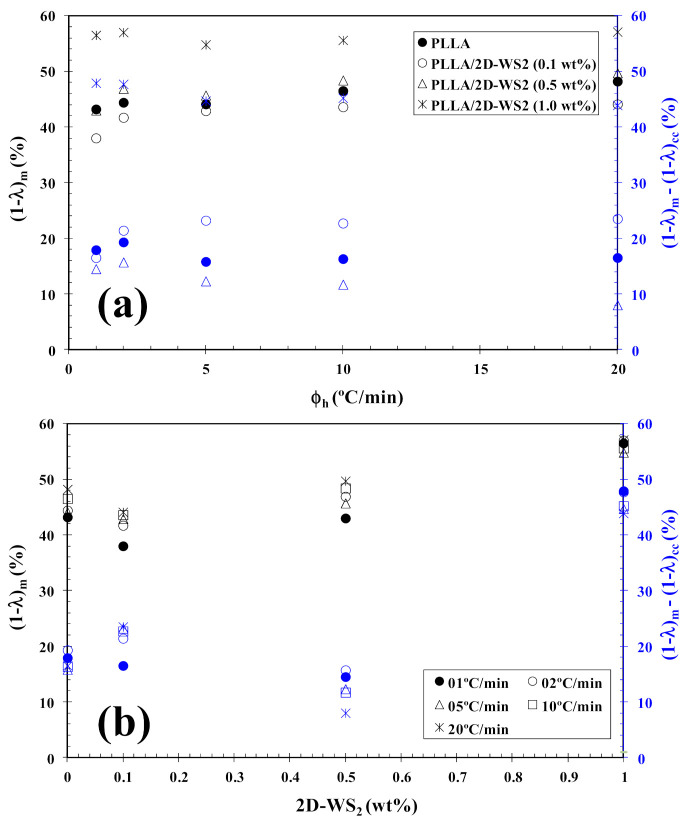
Variation of the cold-crystallization temperature (T_cc_) for PLLA/2D-WS_2_ nanocomposites with (**a**) heating rate and (**b**) composition.

**Figure 5:**
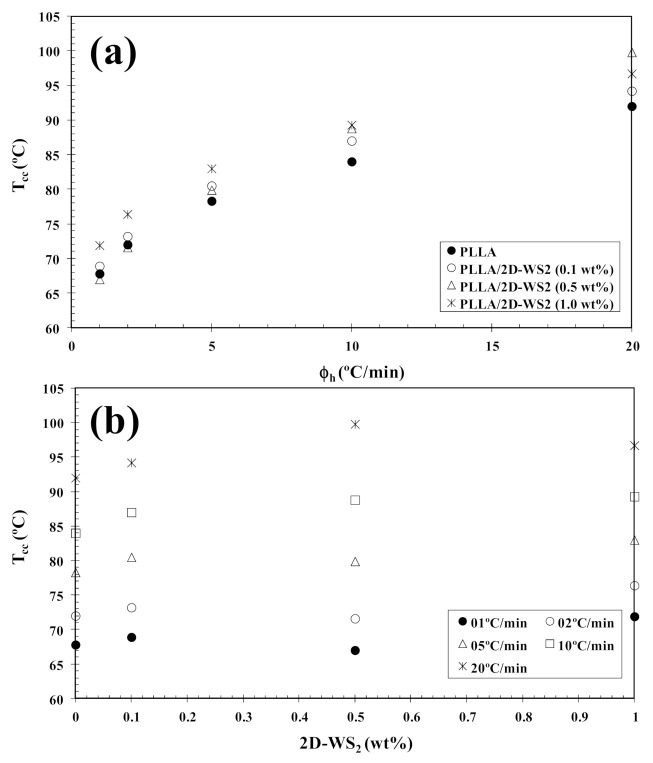
Variation of the melting crystallinity (1−λ)_m_ of PLLA/2D-WS_2_ nanocomposites with (**a**) heating rate and (**b**) 2D-WS_2_ concentration; inset is the difference between the overall crystallinity change derived from the difference between endotherm and exotherm, Δ(1−λ) = (1−λ)_m_ − (1−λ)_cc_, for PLLA/2D-WS_2_ nanocomposites.

In tune with these observations, Figure 4 summarizes the variation of T_cc_ with heating rate and composition. In particular, the addition of 2D-WS_2_ increases the cold-crystallization temperature of PLLA and, therefore, the nucleation of PLLA crystals is retarded by the WS_2_ nanosheets. This effect is independent of the heating rate used. On the other hand, with increasing heating rate, (1−λ)_m_ shifts towards higher values, and the melting process is enhanced for both neat PLLA and its nanocomposites. In the same way, the addition of 2D-WS_2_ apparently enhances (1−λ)_m_ of PLLA when a low heating rate is used. For more clarity, Figure 5 summarizes the variation of (1−λ)_m_ with heating rate and composition. The data presented also show the difference between the overall crystallinity change derived from the difference between endotherm and exotherm, Δ(1−λ) = (1−λ)_m_ − (1−λ)_cc_, for neat PLLA and its nanocomposites, which can be used to highlight the recrystallization ability of the PLLA crystals. At lower ∆(1−λ), the reorganization of the PLLA crystals is more difficult. Thus, increasing the heating rate allows less time for the molten materials to reorganize into new crystals, thus lowering Δ(1−λ) (Figure 5a), and hence, the reorganization process is largely inhibited. In the case of the nanocomposites, the role of 2D-WS_2_ on the variation of the Δ(1−λ) values of PLLA appears to be more relevant at a high nanosheet content (1.0 wt%), improving the recrystallization ability of the PLLA crystals. However, in our case, we cannot exclude the nucleation capacity of 2D-WS_2_ during cooling (this will be discussed later), which may induce the generation of new crystals of PLLA in the nanocomposites. That is, the difference ∆(1−λ) for nanocomposites with 1.0 wt% of 2D-WS_2_ may involve both nucleation during cooling as well as the reorganization ability of the PLLA crystals during subsequent heating.

**Figure 6:**
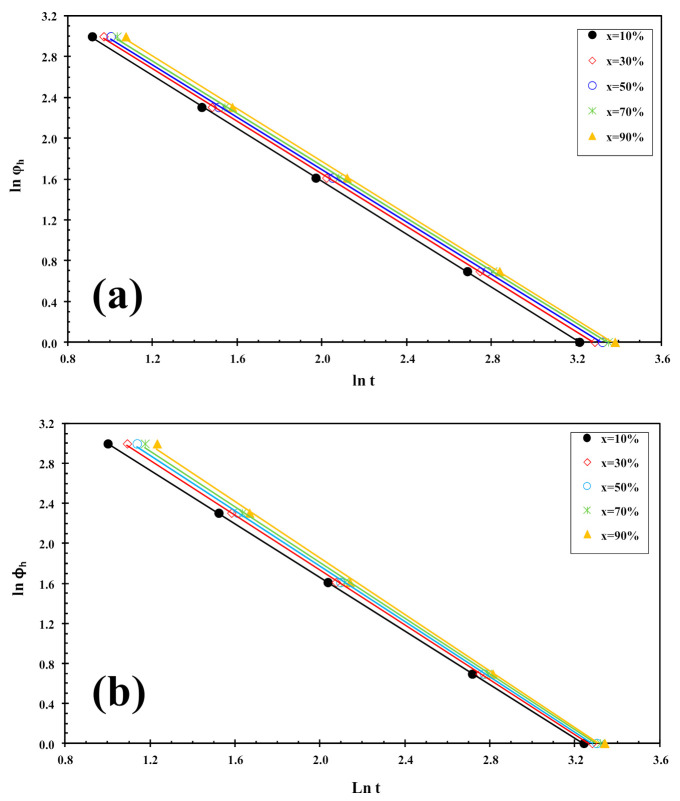
Liu plots for cold-crystallization of (**a**) PLLA and (**b**) PLLA/2D-WS_2_ (0.5 wt%).

The kinetics of cold-crystallization can be described using an alternative model recently proposed by Liu et al. [34]. By combining the Avrami [35,36,37] and Ozawa [38] equations, the Liu model has been proved to be suitable and convenient to handle the non-isothermal crystallization of polymer nanocomposites [39]. The final expression of their model can be written as follows:

(5) $$\ln\varphi =\mathrm{lnf}\left(T\right)-\alpha \mathrm{lnt}$$

where f(T) = [k′(T)/k]^1/m^ refers to the value of cooling rate chosen at unit crystallization time, when the system has a certain degree of crystallinity, α is the ratio of the Avrami exponents to Ozawa exponents (i.e., α = n/m), and ϕ_h_ is the heating rate. Plotting lnϕ_h_ against ln t at a given degree of conversion yielded a linear representation, as shown in Figure 6. The values of f(T) and α are listed in Table 1, from which one can read that the values of f(T) increase systematically with increasing relative crystallinity, indicating that at unit crystallization time, a higher cooling rate should be required to obtain a higher degree of crystallinity, while the values of the parameter a are almost constant (1.2–1.3). A similar trend was observed for PLLA/2D-WS_2_ nanocomposites. However, in the case of the values of f(T) obtained for PLLA/2D-WS_2_ nanocomposites, the results indicate that no relationship can be drawn between the f(T) values for PLLA and the concentration of 2D-WS_2_. This fact suggests that the cold-crystallization behavior of PLLA may be controlled by the crystallinity developed at room temperature after the cooling process rather than the concentration of 2D-WS_2_ in the PLLA nanocomposites. For these nanocomposites, it is difficult to analyze the cold-crystallization process with a single equation since there are many parameters that must be considered simultaneously in the Liu model (i.e., auto-nucleation process, crystallinity developed before the cold-crystallization, etc.).

### 3.3. Non-Isothermal Melt-Crystallization

**Figure 7:**
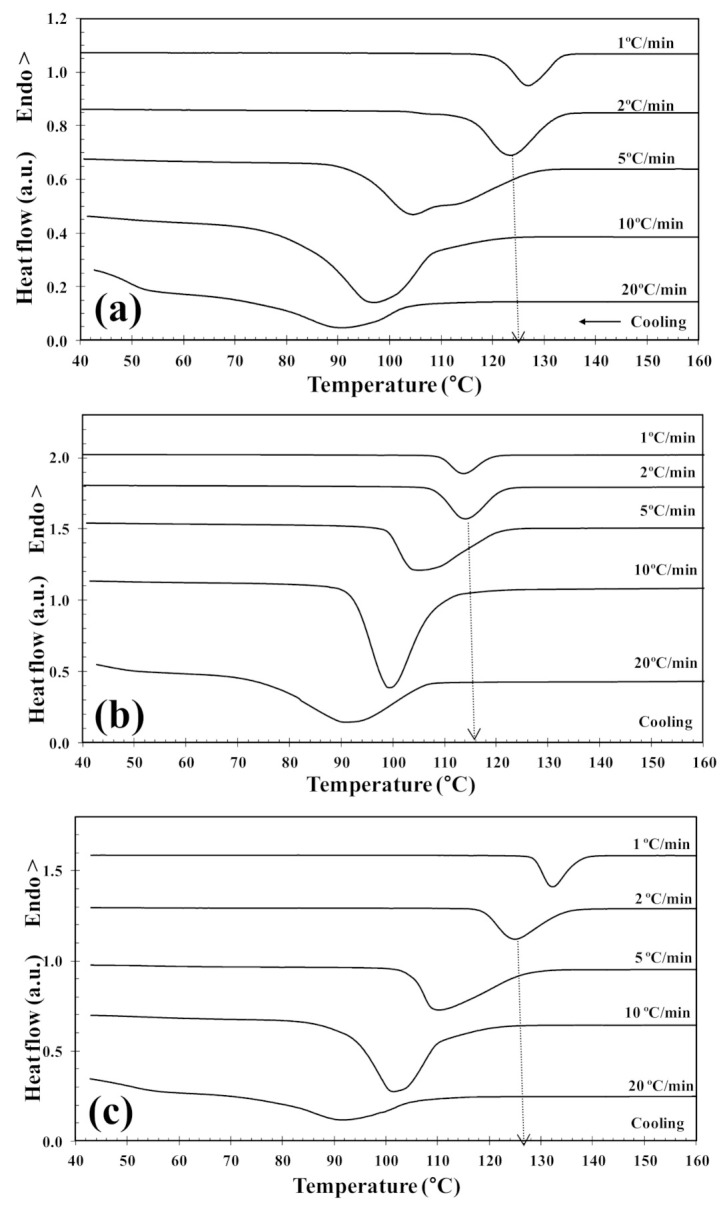
DSC melt-crystallization thermograms of (**a**) PLLA and PLLA/2D-WS_2_ nanocomposites with nanofiller loadings of (**b**) 0.5 and (**c**) 1.0 wt%.

**Figure 8:**
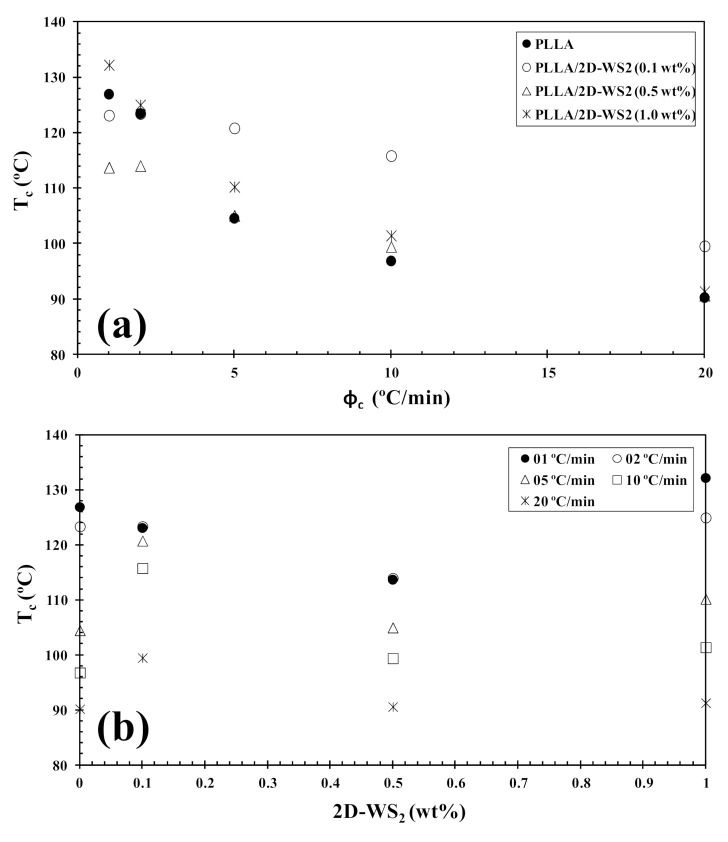
Variation of the melt-crystallization temperature (T_c_) for PLLA/2D-WS_2_ nanocomposites with (**a**) cooling rate and (**b**) composition.

The non-isothermal melt-crystallization behavior of PLLA and the PLLA/2D-WS_2_ nanocomposites was investigated as this corresponds to the type of temperature changes that might occur in industrial applications. Figure 7 shows the effect of cooling rate and 2D-WS_2_ concentration on the non-isothermal crystallization behavior of the PLLA/2D-WS_2_ nanocomposites, with the specific crystalline parameters of all samples listed in Table 2. As the cooling rate increases, the crystallization exotherm broadens and shifts to lower temperatures for PLLA and PLLA/2D-WS_2_ nanocomposites, indicating that the lower the cooling rate, the earlier crystallization occurs. However, the most relevant observation was the influence of 2D-WS_2_ concentration on the crystallization temperature of PLLA for a particular cooling rate. The addition of low concentrations of 2D-WS_2_ (i.e., 0.1 and 0.5 wt%) reduces the melt-crystallization temperature of PLLA, which may imply that the nucleation of PLLA crystals is retarded by the WS_2_ nanosheets. This observation is reproducible for nanocomposites crystallized at different cooling rates, as shown in Figure 8.

**Figure 9:**
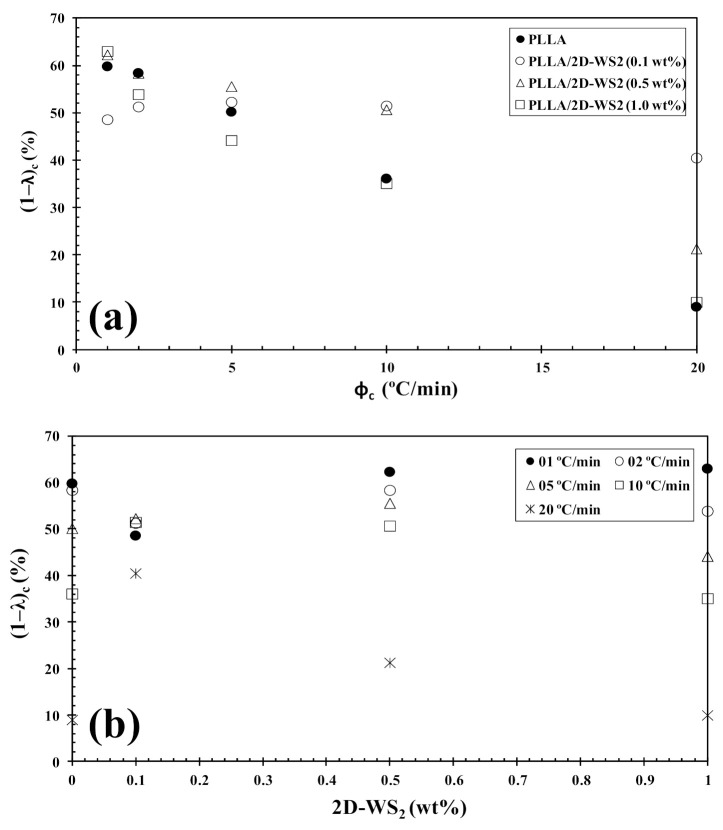
Variation of the crystallinity (1−λ)_c_ for PLLA/2D-WS_2_ nanocomposites with (**a**) cooling rate and (**b**) composition.

However, the further addition of 2D-WS_2_ causes a drastic change from retardation to promotion of the crystallization of PLLA. In a similar manner to the 2D-WS_2_ nanosheets, the addition of inorganic fullerenes (IF-WS_2_) to polyphenylene sulfide (PPS) was also found to exert significant influence on the crystallization kinetic of PPS, but in ways unexpected for polymer nanocomposites [39]. In contrast, 2D-WS_2_ has been shown to reduce the crystallization rate of melt-processable PLLA nanocomposites at any concentration [26]. This discrepancy is likely related to several factors, including the nucleation efficiency (NE) and morphology of the filler, its surface energy, roughness, and state of dispersion within the matrix as well as on the filler’s ability to form the critical nucleus [22,23,24,39]. The addition of 2D-WS_2_ apparently enhances (1-λ)_c_ of PLLA when a high cooling rate is used. In particular, the role of 2D-WS_2_ on the variation of the (1-λ)_c_ values of PLLA appears to be less relevant for a higher nanosheet content (1.0 wt%) (Figure 9).

**Figure 10:**
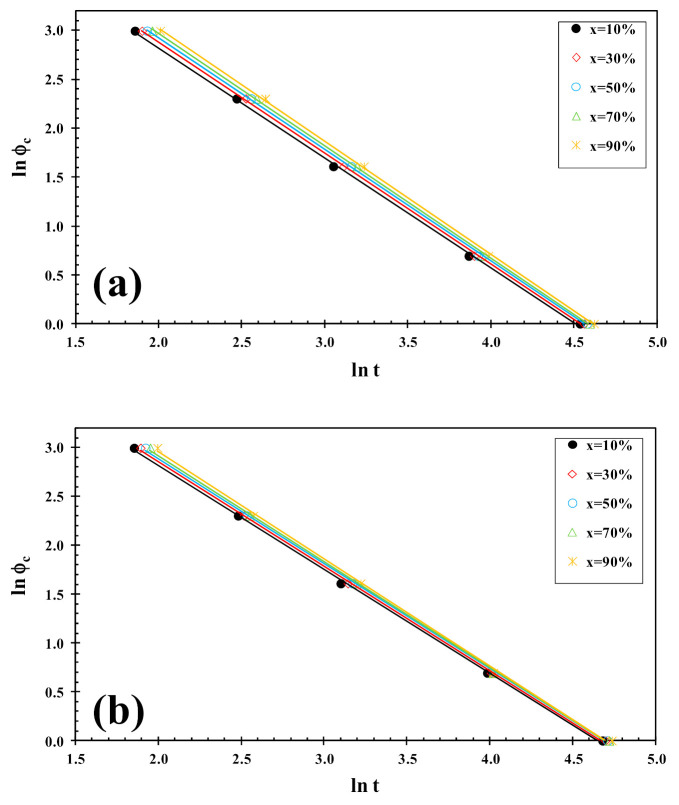
Liu plots for melt-crystallization of (**a**) PLLA and (**b**) PLLA/2D-WS_2_ (0.5 wt%).

On the other hand, the Liu analysis of the PLLA/2D-WS_2_ nanocomposites was performed in order to confirm the last dynamic melt-crystallization observations. For example, Figure 10 shows the plots of lnϕ_c_ against ln t at a given degree of conversion (x), which yield to a linear representation of Equation (5). This indicates that the Liu model provides a satisfactory description to the melt-crystallization behavior of PLLA/2D-WS_2_ nanocomposites. The kinetic parameters, lnf(T) and α, which are derived from the slope and the intercept of those lines, are listed in Table 2. It can be observed that the value of f(T) for neat PLLA is lower that for the nanocomposites with low concentrations (i.e., less than or equal to 0.5 wt%), which indicates that the nanocomposites require a higher heating rate to approach an identical degree of crystalline transformation. In other words, the crystallization rate of the nanocomposites is lower than that of neat PLLA. However, the further addition of 2D-WS_2_ causes a change from retardation to promotion of the crystallization of PLLA. It is also noted that the dynamic crystallization is difficult to be analyzed with a single equation since there are a lot of parameters that have to be considered simultaneously. The importance of this method is that it correlates the cooling rate to temperature, time and morphology.

**Figure 11:**
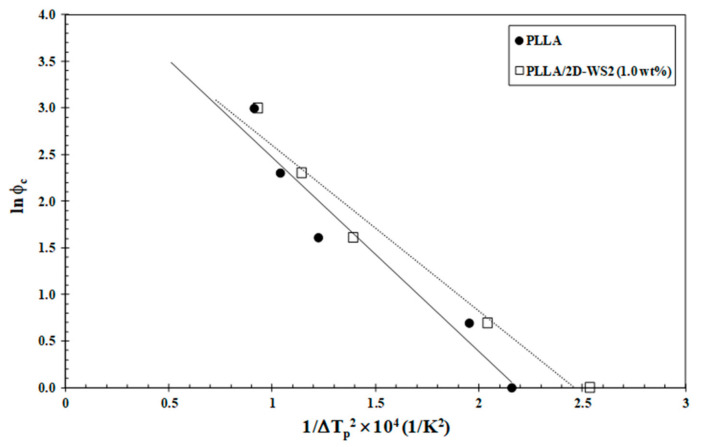
Dobreva plots for evaluating nucleation activity of 2D-WS_2_ in PLLA/2D-WS_2_ (1.0 wt%) nanocomposites.

Since the crystallization behavior of PLLA was influenced by 2D-WS_2_ as well as its loading, a quantitative estimate of the nucleation capability of 2D-WS_2_ in the PLLA matrix was undertaken. Dobreva and Gutzow [40,41] suggested a simple method for calculating the nucleation activity (φ) of foreign substrates in polymer melt, whose value varies from 0 to 1, corresponding to extremely active and inert foreign substrates, respectively. In particular, the more active the nucleator is, the lower the value of φ should be. According to this model, the nucleation activity can be calculated from the ratio:

(6) $$\varphi =\frac{B^{*}}{B}.$$

where B is a parameter for the pristine polymer, and B* is forthe polymer/nucleator system. B and B* can both be experimentally determined from the slope of the following equation:

(7) $$\ln\varphi =A-\frac{B({\mathrm{orB}}^{*})}{\Delta T_{p}^{2}}$$

where ϕ_c_ is the cooling rate, A is a constant, ΔT_p_ is the supercooling (T_m_−T_c_), T_m_ is the melting point temperature and T_c_ is the crystallization peak temperature. A linear relationship was obtained for both neat PLLA and PLLA/2D-WS_2_ (1.0 wt%), as can be observed in Figure 11. The values of B and B* are obtained from the slope of the fitted lines, and the nucleation activity (φ) is calculated from this ratio. The value of nucleation activity for PLLA/2D-WS_2_ (1.0 wt%) is 0.86. This means that 2D-WS_2_ is causing the nucleation of PLLA. It is presumed that PLLA crystals might grow on the surface of 2D-WS_2_ by an epitaxial mechanism, as suggested in the previous literature for PLLA and other fillers. However, additional information is still needed for an in-depth understanding of the exact nucleation mechanism [24,42]. This result of the nucleating activity of 2D-WS_2_ on the PLLA matrix justifies the increase in the melt-crystallization temperature of PLLA in the nanocomposites with 1.0 wt% of 2D-WS_2_ (Figure 7 and Figure 8).

## 4. Conclusions

In the present investigation, inorganic nanosheets (2D-WS_2_) were successfully dispersed into a PLLA matrix through simple solution blending, as confirmed by SEM and TEM analysis. The results obtained from differential scanning calorimetry analysis reveal that the presence of 2D-WS_2_ modifies the crystallization behavior of PLLA, whilst it does not alter its crystallization mechanism. The cold-crystallization rate of PLLA is found to be greatly reduced upon the addition of increasing 2D-WS_2_ loadings due to the physical barrier action of the nanosheets. This effect is independent of the heating rate used. On the other hand, the heating of neat PLLA and PLLA/2D-WS_2_ nanocomposites led to the appearance of complex double-melting peaks due to a melt-recrystallization mechanism. In particular, the difference in the apparent crystallinity ∆(1−λ) was found to be an effective method to highlight the recrystallization ability of the PLLA crystals. The quantitative analysis showed that increasing the heating rate can effectively inhibit the reorganization process of PLLA, and a further increase in the 2D-WS_2_ concentration led to a marked improvement in the recrystallization of PLLA. Furthermore, the method developed by Liu et al. could successfully describe the complex melt-crystallization kinetics of the PLLA/2D-WS_2_ nanocomposites occurring during continuous cooling. The parameter f(T), which has a physical and practical significance, suggested that the increase in the 2D-WS_2_ content caused a change from retardation to promotion of the crystallization of PLLA. In particular, the addition of 1.0 wt% of 2D-WS_2_ concentration leads to an enhancement of the transport ability of polymer chains due to the nucleation role of 2D-WS_2_, thus increasing the crystallization rate of PLLA. In the same way, the results of the nucleation activity using the Dobreva model confirmed the unique dependence of the crystallization behavior of PLLA on composition. These observations have considerable practical significance for the future sustainable, economic and effective technological utilization of PLLA, as it will enable the development of novel biopolymer nanocomposite materials.

## Figures and Tables

**Table 1:** Cold-crystallization and melting parameters of the neat PLLA and PLLA/2D-WS_2_ nanocomposites.

2D-WS_2_ (wt%)	ϕ_h_ (°C/min)	T_cc_ (°C)	(1−λ)_cc_ (%)	T_m1_ (°C)	T_m2_ (°C)	(1−λ)_m_ (%)	x ^a^ (%)	α ^a^	*f*(T) ^a^
	1	67.8	25.3	-	154.0	43.2	10	1.30	4.18
	2	72.0	25.1	141.6	154.4	44.4	30	1.29	4.18
0.0	5	78.3	28.3	143.7	154.5	44.1	50	1.30	4.27
	10	84.0	30.2	143.8	154.1	46.5	70	1.29	4.30
	20	92.0	31.7	144.7	154.3	48.2	90	1.29	4.36
	1	68.9	21.5	-	149.9	38.0	10	1.31	4.07
	2	73.2	20.3	-	150.2	41.7	30	1.29	4.21
0.1	5	80.5	19.7	-	150.8	42.9	50	1.29	4.30
	10	87.0	20.9	-	151.5	43.6	70	1.30	4.30
	20	94.2	20.6	-	151.7	44.1	90	1.32	4.48
	1	67.0	28.5	-	152.9	43.0	10	1.34	4.34
	2	72.6	31.2	-	153.3	46.9	30	1.37	4.47
0.5	5	79.9	33.4	-	153.5	45.7	50	1.38	4.54
	10	88.8	36.7	-	153.5	48.4	70	1.39	4.60
	20	99.8	41.7	141.2	154.1	49.7	90	1.41	4.69
	1	71.9	8.6	164.4	167.8	56.5	10	1.29	4.14
	2	76.4	9.3	165.6	168.6	57.0	30	1.28	4.26
1.0	5	83.0	10.1	166.1	169.0	54.8	50	1.26	4.31
	10	89.3	10.4	166.4	169.1	55.6	70	1.26	4.35
	20	96.7	13.2	159.8	169.5	57.1	90	1.25	4.40

^a^ Crystallization Parameters Calculated Using Liu’s Equation.

**Table 2:** Melt-crystallization and melting parameters of the neat PLLA and PLLA/2D-WS_2_ nanocomposites.

2D-WS_2_ (wt%)	ϕ_c_ (°C/min)	T_c_ (°C)	(1−λ)_c_ (%)	T_cc_ (°C)	(1−λ)_cc_ (%)	T_m1_ (°C)	T_m2_ (°C)	(1−λ)_m_ (%)	*x*^a^ (%)	*α* ^a^	*f*(T) ^a^
0	1	126.9	59.8	-	-	-	153.5	64.9	10	−1.12	5.07
	2	123.4	58.4	-	-	-	166.0	64.8	30	−1.14	5.15
	5	104.5	50.2	-	-	167.4	171.9	58.0	50	−1.14	5.21
	10	96.8	36.1	88.4	2.7	-	171.9	53.0	70	−1.15	5.23
	20	90.2	9.0	94.2	24.2	-	172.1	50.2	90	−1.16	5.34
0.1	1	123.1	45.2	-	-	-	152.2	51.1	10	−1.05	4.79
	2	123.4	47.7	-	-	-	156.9	57.0	30	−1.06	4.86
	5	120.8	48.6	-	-	-	160.5	58.6	50	−1.07	4.91
	10	115.8	47.9	-	-	-	163.3	59.5	70	−1.08	4.95
	20	99.5	37.7	-	-	165.0	170.1	56.0	90	−1.08	5.01
0.5	1	113.7	57.9	-	-	148.3	156.0	66.9	10	−1.06	4.94
	2	114.0	54.3	-	-	152.8	160.6	66.7	30	−1.07	5.00
	5	105.0	51.7	-	-	158.4	165.3	66.2	50	−1.08	5.04
	10	99.4	47.1	-	-	162.0	169.4	64.6	70	−1.08	5.08
	20	90.6	19.8	82.5	14.7	154.8	170.4	57.7	90	−1.10	5.15
1	1	132.2	63.0	-	-	-	170.3	68.6	10	−1.13	5.08
	2	125.0	53.9	-	-	-	172.2	59.9	30	−1.14	5.15
	5	110.2	44.2	-	-	-	172.5	50.9	50	−1.15	5.20
	10	101.4	35.1	-	-	-	176.2	47.5	70	−1.16	5.25
	20	91.3	10.0	99.7	17.4	-	175.6	42.6	90	−1.17	5.34

^a^: is the ratio of the Avrami exponents to Ozawa exponents.

## Data Availability

Not applicable.

## Supplementary Materials

The following are available online at https://www.mdpi.com/article/10.3390/polym13132214/s1, Figure S1: Initiator used for the synthesis of PLLA, Figure S2: Representative 1H NMR spectrum of PLLA obtained by 1, Figure S3: Representative 13C{1H}-NMR spectrum of PLLA obtained by 1, Figure S4: Representative GPC profile of PLLA prepared by 1.
