# Supplementary material for: The Effect of WS2 Nanosheets on the Non-Isothermal Cold- and Melt-Crystallization Kinetics of Poly(l-lactic acid) Nanocomposites
Source: Polymers (Basel). 2021 Jul 5;13(13):2214. doi: 10.3390/polym13132214 (PMC8271659; doi:10.3390/polym13132214)
Supplement: Supplementary file 1 [file polymers-13-02214-s001.zip › polymers-1272997-supplementary.pdf]

Supporting Information

The Effect of WS_2 Nanosheets on the Non-Isothermal Cold- and Melt-Crystallization Kinetics of Poly(L-lactic acid) Nanocomposites

Mohammed Naffakh[1], Pablo Rica[1], Carmen Moya-Lopez[2,3], José Antonio Castro-Osma[2,4], Carlos Alonso-Moreno[2,4], Diego A. Moreno[1,2]

[1]Escuela Técnica Superior de Ingenieros Industriales, Universidad Politécnica de Madrid (ETSII-UPM), José Gutiérrez Abascal 2, 28006 Madrid, Spain pablo.rica.mozo@alumnos.upm.es (P.R.); diego.moreno@upm.es (D.A.M.)

[2]Facultad de Farmacia, Universidad de Castilla-La Mancha (FF-UCLM), Avda. Dr. José María Sánchez Ibánez s/n, E-02071 Albacete, Spain

carmen.moya-lopez-pelaez@univ-lorraine.fr (C.M.P); JoseAntonio.castro@uclm.es (J.A.C-O.); carlos.amoreno@uclm.es (C.A.M.)

[3]LMOPS, CentraleSupelec, University of Lorraine, 2 rue E. Belin, 57070 Metz, France.

[4]Centro Regional de Investigaciones Biomédicas. Unidad NanoCRIB, Albacete 02008, Spain.

* Correspondence: mohammed.naffakh@upm.es

1. Synthesis of [$AlEt_2\{\kappa^2$-bpzbe}] (1)

In 250 cm^3 Schlenk tube, bpzbeH (1.0 g, 3.4 mmol) was dissolved in dry toluene (70 mL) and heated to 50 °C. A solution of $AlEt_3$ (1 M in hexane, 3.4 mL, 3.4 mmol) was added and the reaction mixture was stirred at this temperature for 2 h. Removal of the volatiles under reduced pressure yielded **1** as a white solid. The product was washed with n-hexane (25 mL) and recrystallized from toluene at −26 °C to give compound **1** as a white solid.

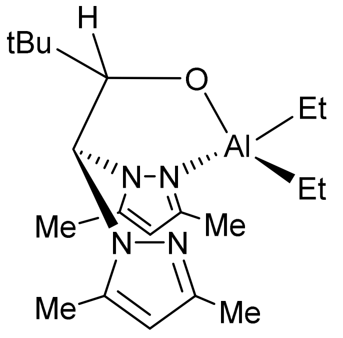

Initiator 1

Figure S1. Initiator used for the synthesis of PLLA.

2. Poly(L-lactic acid) Characterization

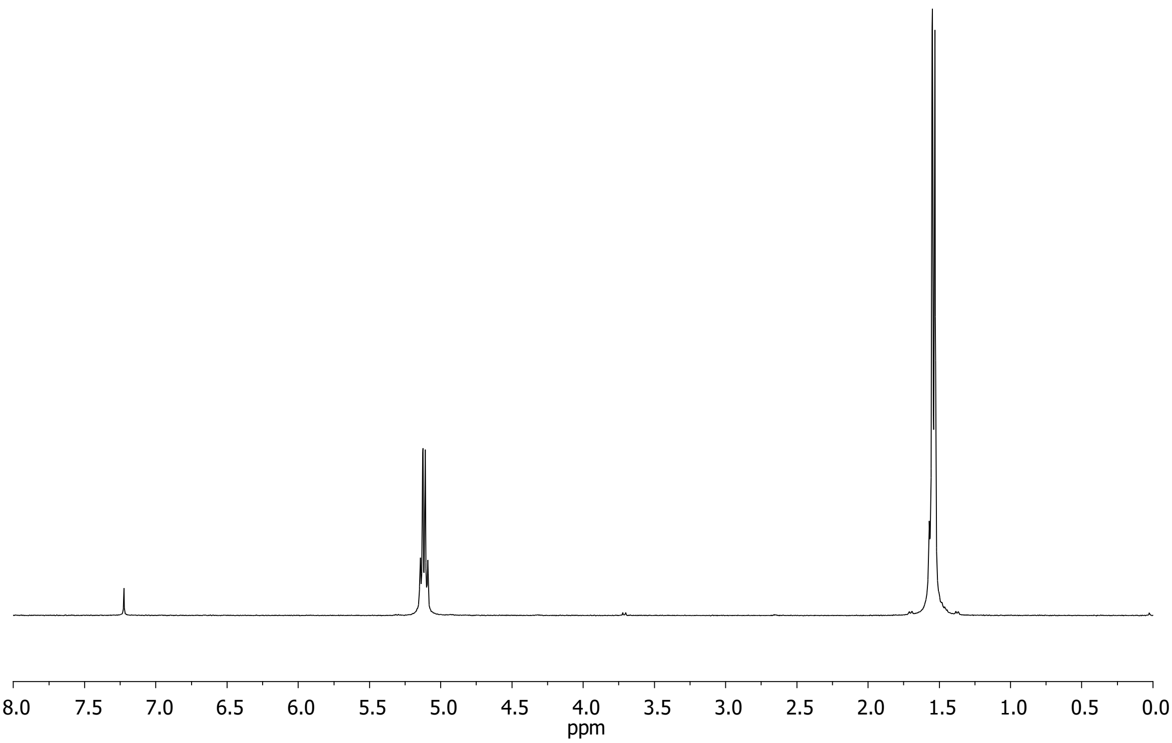

Figure S2. Representative ^{1}H NMR spectrum of PLLA obtained by **1**.

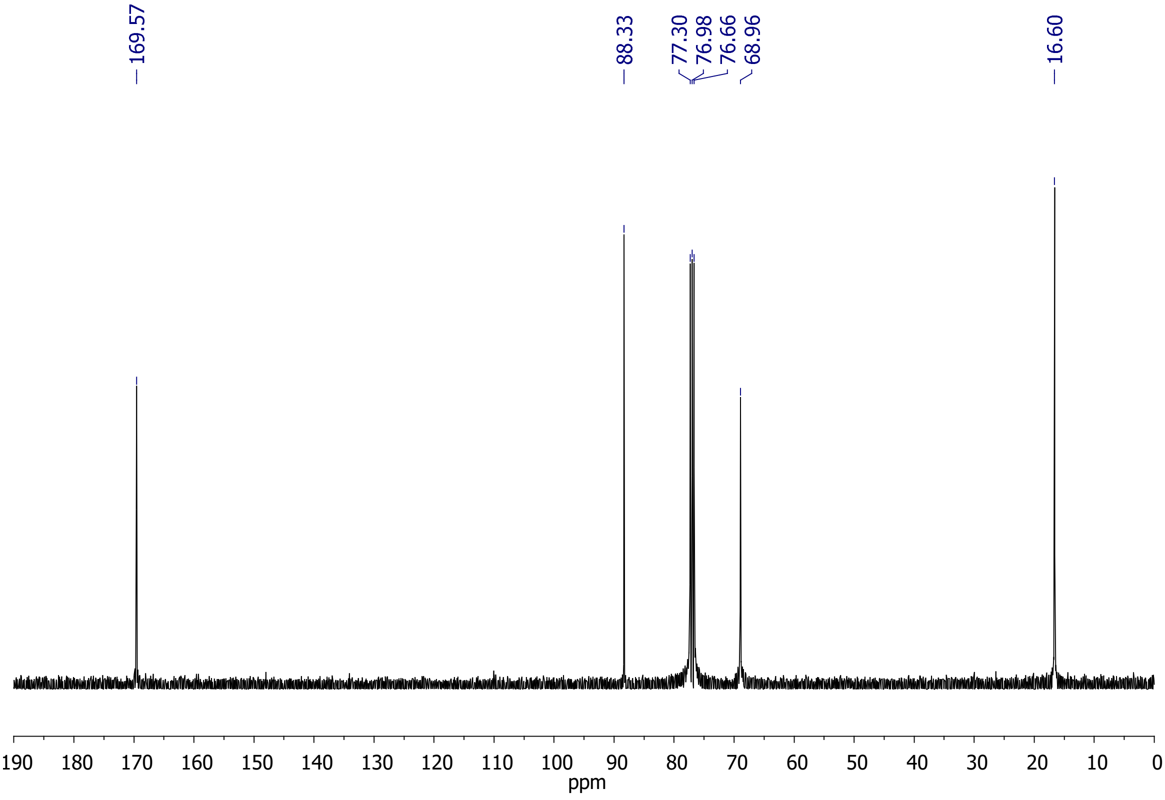

Figure S3. Representative $^{13}C\{^{1}H\}$-NMR spectrum of PLLA obtained by **1**.

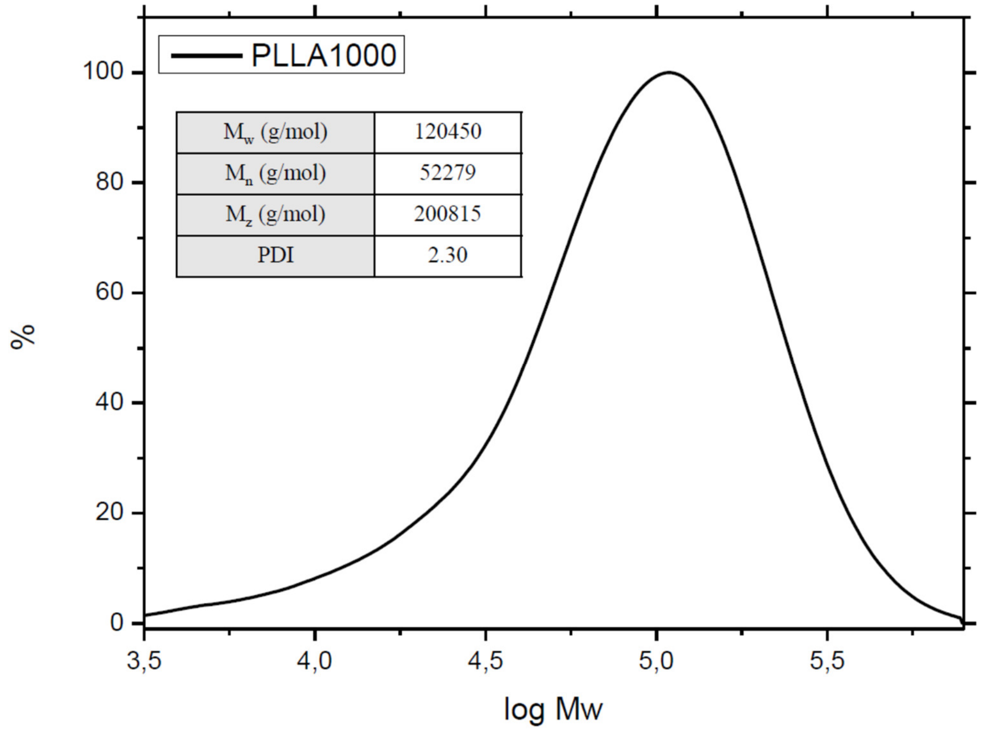

Figure S4. Representative GPC profile of PLLA prepared by **1**.
